# Large-scale molecular dynamics simulation: Effect of polarization on thrombin-ligand binding energy

**DOI:** 10.1038/srep31488

**Published:** 2016-08-10

**Authors:** Li L. Duan, Guo Q. Feng, Qing G. Zhang

**Affiliations:** 1 School of Physics and Electronics, Shandong Normal University, Jinan 250014, China

## Abstract

Molecular dynamics (MD) simulations lasting 500 ns were performed in explicit water to investigate the effect of polarization on the binding of ligands to human α-thrombin based on the standard nonpolarizable AMBER force field and the quantum-derived polarized protein-specific charge (PPC). The PPC includes the electronic polarization effect of the thrombin-ligand complex, which is absent in the standard force field. A detailed analysis and comparison of the results of the MD simulation with experimental data provided strong evidence that intra-protein, protein-ligand hydrogen bonds and the root-mean-square deviation of backbone atoms were significantly stabilized through electronic polarization. Specifically, two critical hydrogen bonds between thrombin and the ligand were broken at approximately 190 ns when AMBER force field was used and the number of intra-protein backbone hydrogen bonds was higher under PPC than under AMBER. The thrombin-ligand binding energy was computed using the molecular mechanics Poisson-Boltzmann surface area (MM/PBSA) method, and the results were consistent with the experimental value obtained using PPC. Because hydrogen bonds were unstable, it was failed to predict the binding affinity under the AMBER force field. Furthermore, the results of the present study revealed that differences in the binding free energy between AMBER and PPC almost comes from the electrostatic interaction. Thus, this study provides evidence that protein polarization is critical to accurately describe protein-ligand binding.

The first molecular dynamics (MD) simulation of protein began in 1977 by simulating bovine pancreatic trypsin inhibitor (BPTI), and this work introduced a new era of “structure to function” in biophysics^1^. MD simulation is a powerful tool for revealing important biological phenomena and understanding the mechanisms underlying the interactions of biological macromolecules, such as enzyme reactions, protein ligand binding, protein folding, protein association, protein hydration and ion channels mechanisms, etc.^2^,^3^,^4^,^5^,^6^,^7^,^8^,^9^,^10^,^11^,^12^,^13^,^14^ And it provides detailed information of dynamics behaviors of proteins at atomic level. The simulation accuracy is dependent on two factors: simulation time (conformational sampling should be throughout the entire phase) and accuracy of the force field. With the development of simulation methodologies and computational resources, the conformational sampling bottleneck is gradually diminishing. MD simulation has recently been extended to greater conformational changes and longer time scales ranging from femtoseconds to milliseconds and they also realize the current microcosms-to-mesoscopic transitions in space. Thus, the accuracy of the employed force field is the dominant factor controlling outcome of MD simulation.

Although the current force fields including AMBER, CHARMM, GROMACS and OPLS have achieved great success in the past two decades^15^,^16^,^17^,^18^,^19^,^20^, while fundamental limitations affect their applications. They are fitted to high-level quantum mechanical calculations or experimental data for individual amino acids in the gas phase or a homogeneous environment^21^. Thus, these force fields are specific to amino-acids or similar to mean fields and cannot accurately describe the electrostatic interactions in highly inhomogeneous protein-specific environments. Electrostatic interaction plays a critical role in studies concerning the properties of all types of biological macromolecules^22^,^23^,^24^,^25^,^26^ and the electronic polarization effect is remarkable in polar dielectrics, particularly in water. The lack of electronic polarization of proteins in currently popular force fields, leads to uncertainties regarding the accuracy and reliability of MD simulation results.

In order to overcome these force fields’ deficiencies, many attempts have been made to develop polarizable force fields^27^,^28^. Although some polarizable force fields, such as the fluctuating charge model, Drude oscillator, and induced multi-pole^29^,^30^,^31^,^32^,^33^,^34^,^35^,^36^,^37^, have been improved in recent years, while the practical applications of these models remain uncommon because of their high computational costs, and the validity of the results remains limited because of difficulties in tuning the parameters^38^. So it is still unpractical to run large-scale MD simulation using those polarizable force fields. The development of polarizable force fields is lagging, and thus, the need for a reasonably accurate and efficient force field to accurately represent the electronic polarization state of a protein in a given conformation is becoming increasingly critical.

Recently, a new charge scheme for protein dynamics, termed the polarized protein specific charge (PPC) was developed to accurately represent the electrostatic interactions of proteins^39^,^40^,^41^,^42^,^43^,^44^,^45^,^46^,^47^,^48^,^49^,^50^,^51^,^52^,^53^,^54^,^55^,^56^,^57^,^58^,^59^,^60^,^61^,^62^,^63^,^64^. In PPC, the partial atomic charges of proteins are determined through quantum chemistry calculations of proteins in solvents using a fragment approach combined with a continuum solvent model^39^, constituting a straightforward method for incorporating the polarization effects through charge fitting to the electrostatic potential. Thus, PPC provides a more reliable description of the protein structure and dynamics because it contains the proper electronic polarization effect. During MD simulation, the atomic charges of traditional force field are replaced by PPC while maintaining all other parameters. Therefore, the PPC does not add any dynamic complications. The advantage of PPC over traditional force fields was demonstrated through successful applications, such as pKa prediction, NMR coupling, protein-ligand binding affinity, protein folding, native structure stabilization, protein-protein interaction, ect^64^, which was in agreement with many other studies^38^. Especially, secondary structures were more stable and better preserved using PPC force field than unpolarizable AMBER force filed. And some studies had indicated that PPC force field could avoid incorrect deformation of protein structure and kept those important structural properties of protein. And the inclusion of the polarization effect resulted in more dynamically stable structures. Those simulation structures under PPC force filed were in excellent agreement with experimental observations^40^,^41^. And we also made some comparison against AMBER02 polarizable force field in protein folding simulation. Our results showed that AMBER02 polarizable force gave very similar to that from nonpolarizable AMBER03 force field, which were both failed to fold the helix within the simulation time. However, the protein was successfully folded into the native structure using PPC force field^57^.

Human α-thrombin (thrombin) is a multifunctional trypsin-like serine protease involved in some physiological processes in the human body and the final enzyme in the blood coagulation cascade. Thrombin is a complicated protein, comprising 276 amino acid residues. The two primary functions are the cleavage of fibrinogen to release fibrin which forms the fibrin gel of a hemostatic plug or a pathologic thrombus, and the activation of platelets through the thrombin receptor^65^,^66^,^67^. As a procoagulant enzyme, the generation of thrombin is self-regulated through the activation of coagulation factors V and VIII resulting in the formation of a burst of thrombin^68^,^69^,^70^,^71^. The enzyme interacts with multiple procoagulant substrates to mediate both fibrin clotting and platelet aggregation at the site of injury^72^,^73^,^74^. In contrast, in its anticoagulant role, thrombin acts as a negative regulator through the activation of zymogen protein C when bound to thrombomodulin, a receptor on the membranes of endothelia cells, thereby attenuating clot formation^75^,^76^,^77^. The efficiency of the coagulation cascade depends on the balance between pro- and anticoagulants. Because of its dual role, thrombin is a key player in this balance. In addition, this enzyme is a powerful agonist for a variety of cell types and is also involved in many other activities, such as inflammation, wound healing and the regulation of endothelial cell function^78^,^79^. Recent multidisciplinary studies have provided a wealth of information to understand the structure and function of thrombin because this enzyme is fundamentally important in biology and medicine. L86 is an orally bioavailable direct and macrocyclic inhibitor of thrombin with high degrees of potency and selectivity^80^. This inhibitor forms three hydrogen bonds with the SER214 and GLY216 residues in thrombin (here, the numbering of residues is consistent with description of Nantermet *et al*.^80^), making dominant contributions to the stabilization of the thrombin/L86 complex.

In this study, large-scale MD simulations were performed using an AMBER12SB force field and PPC to characterize the interaction dynamics between thrombin and L86. Atomic charges in PPC were fitted to the electrostatic potential around each residue and L86 using high-level quantum mechanics calculations. The time-scale of the MD simulation was as long as 500 ns, which is sufficient for sampling the representative conformations. Although more accurate methods for calculation the binding free energy are FEP (free energy perturbation) or TI (thermodynamic integration), they are both prohibitively expensive and very difficult to converge numerically^81^. So the MM/PBSA method^82^ was used to obtain the free energies of thrombin binding to L86 under AMBER and PPC, respectively, to explicitly investigate the effects of protein polarization. Especially, under PPC force field, these trajectories were generated from MD simulation considering polarizable effect. With these trajectories, the polarized charge was used to calculate the binding free energy using MM/PBSA method. In fact, in our MM/PBSA calculation using PPC force field, the polarization effect had been sufficiently included. In this study, a detailed comparison between the polarizable force field and the standard AMBER force field was shown.

## Results and Discussion

**Figure 1:**
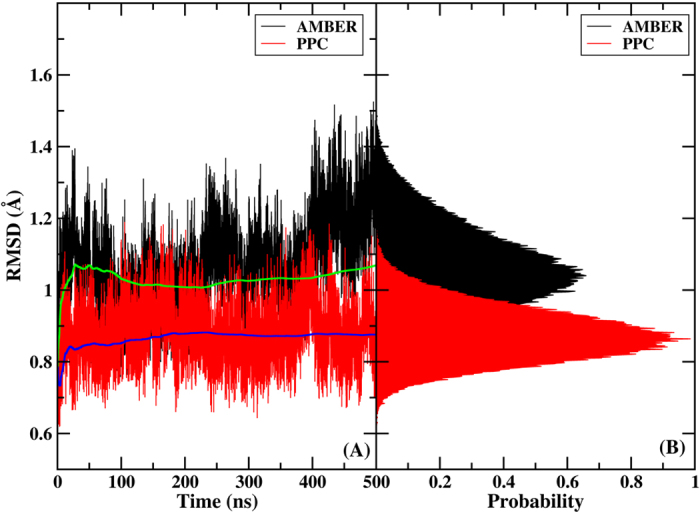
(**A**) Comparison of the RMSDs from two 500 ns simulations based on the AMBER force field (black) and PPC scheme (red). The average RMSD based on the PPC simulation is shown as a blue line. For comparison, the average RMSD based on the AMBER simulation is shown as a green line. (B) Distributions of the RMSDs of AMBER (black) and PPC simulations (red).

Electrostatic interaction plays an important role in the dynamic stability of the protein-ligand complex^47^. Because PPC is obtained from the first principal quantum solvation calculation of protein, it accurately represents the polarized electrostatic state of the protein and it should provide more accurate electrostatic interaction than the AMBER force field. Variations in the root mean square deviation (RMSD) from the crystal structure of the backbone atoms in the 500 ns simulations are shown in Fig. 1A using the AMBER12SB force field and PPC, respectively. Clearly, the final RMSD varied by approximately 1.4 Å in the AMBER simulation. Compared with the native structure obtained from the X-ray diffraction, this result was fairly reasonable. The RMSD was approximately 0.7 Å under PPC, smaller than that under AMBER12SB. The backbone RMSD distributions during the entire simulation were further analyzed in Fig. 1B. The most populated state had an RMSD of 0.8 Å under PPC, and 1.1 Å under the AMBER force field. The root mean square fluctuation (RMSF) was used to illustrate the conformation fluctuation around the average and is a powerful tool for studying the stability of the protein in MD simulation.

**Figure 2:**
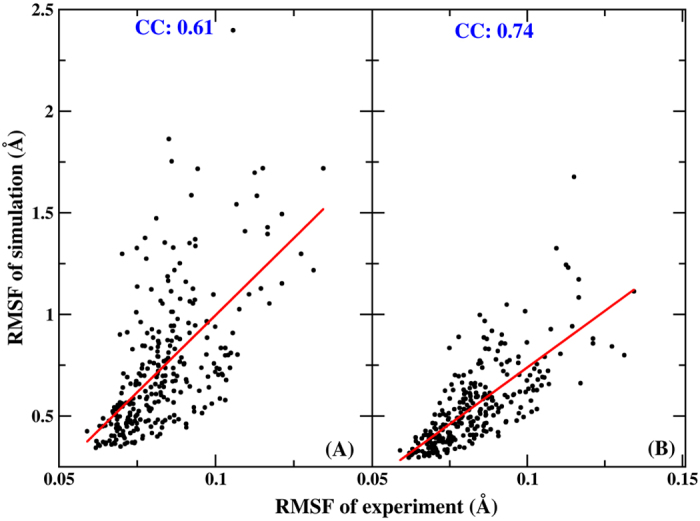
(**A**) The correlation of the Cα RMSF values between X-ray experimental data and the AMBER calculated result. (**B**) The correlation of the Cα RMSF values between X-ray experimental data and the PPC calculated result. CC denotes the correlation coefficient.

The correlation of the Cα RMSFs between the X-ray experimental data and the calculated results using AMBER and PPC plotted in Fig. 2. A good correlation was obtained between the PPC simulation and the experiment data, with a correlation coefficient of 0.74. However, a poor linear relationship was observed for the AMBER simulation, and the correlation coefficient was 0.61. The average Cα RMSFs were 0.74 Å and 0.55 Å using AMBER and PPC, respectively. Therefore, PPC provides a more dynamically stable protein structure, indicating that this force filed was appropriate for the following study.

**Figure 3:**
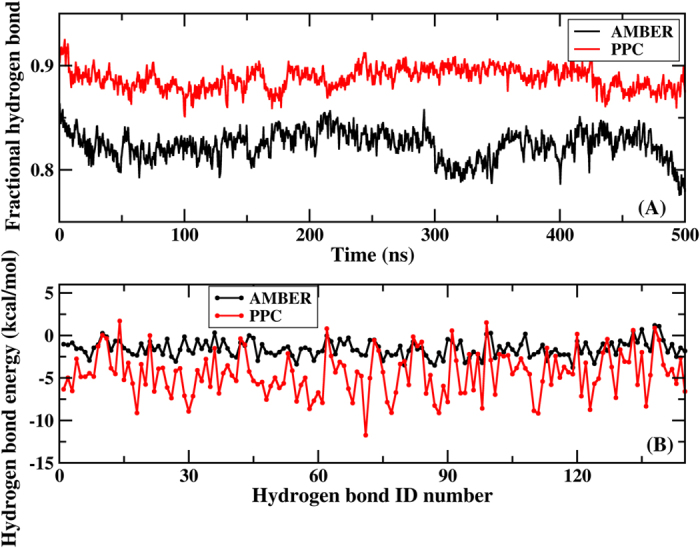
(**A**) Comparison of the fractional backbone native hydrogen bonds every 100 snapshots during MD simulation as a function of time using AMBER (black) and PPC (red). (**B**) Comparison of the average energy of the backbone native hydrogen bonds from MD simulations using AMBER (black) and PPC (red).

The stable three-dimensional structure of a protein is determined by a delicate balance among all types of weak interactions, of which hydrogen bonds are arguably one of the most important. Hydrogen bonds are dominated by electrostatic interactions, and polarization is important in determining the interaction energies of hydrogen bonds. When PPC is employed, the hydrogen bonds (including intra-protein and protein-ligand) should be more stable during the MD simulation. First, we examined the numbers and energies of the intra-protein hydrogen bonds from simulations under AMBER12SB and PPC, respectively. The hydrogen bond length and angle cutoffs were 3.5 Å and 120˚, respectively. A total of 145 backbone hydrogen bonds were detected in the native structure. The fractional backbone native hydrogen bonds present in both the native and simulated structures were divided by the total number in the native structure. Figure 3A illustrates the fractional backbone native hydrogen bonds during MD simulation using AMBER and PPC, respectively, averaged every 100 snapshots. The fractional backbone native hydrogen bonds were obviously higher in PPC than in AMBER, and the final fractional hydrogen bond proportions were approximately 80% and 90% for AMBER and PPC, respectively. These results suggested that PPC was more accurate regarding the stabilization of the intra-protein hydrogen bonds, consistent with previous finding^41^. We next calculated the energy of 145 backbone native hydrogen bonds averaged from the total MD simulation. The Coulomb electrostatic energy (between CO and HN pairs) was used to determine the hydrogen bond energy. Dramatic differences can be observed in Fig. 3B, showing that the hydrogen bond energy under AMBER was smaller than under PPC, reflecting the lack of electronic polarization. The hydrogen bond energy fluctuated by approximately −1.55 kcal/mol under AMBER vs. −4.50 kcal/mol under PPC. Because of the relatively weak hydrogen bond energy, some hydrogen bonds might be easily broken when using an AMBER force field during MD simulation.

**Figure 4:**
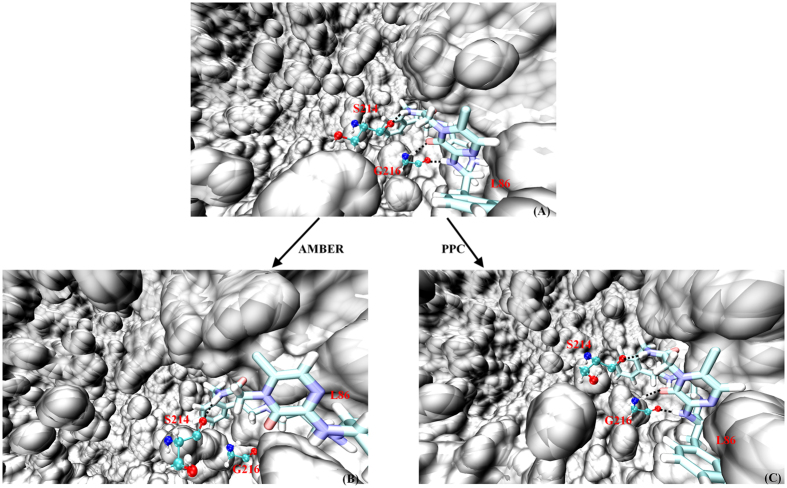
(**A**) The native structure of thrombin and L86, (**B**) the simulated final structure using AMBER, and (**C**) the simulated final structure using PPC. L86 is displayed using stick representation, thrombin is displayed using surface representation and the residues forming hydrogen bonds with L86 are shown as a ball-and-stick model.

**Figure 5:**
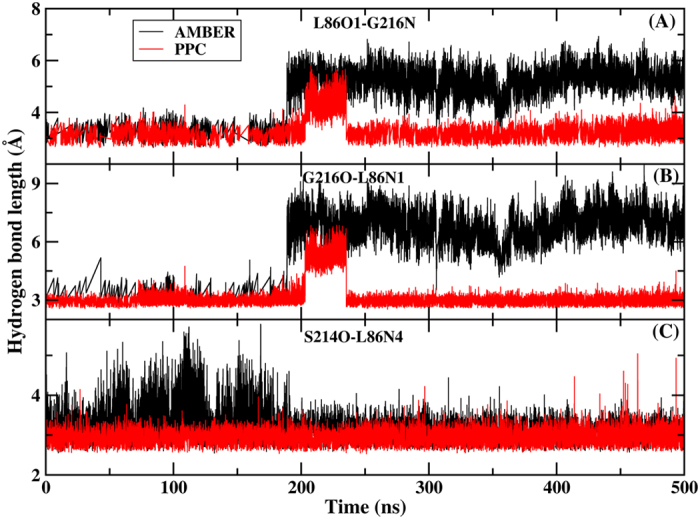
Evolution of the hydrogen bond length between thrombin and L86 under AMBER (black) and PPC (red). (**A**) The hydrogen bond formed between L86O1 and G216N, G216H. (**B**) The hydrogen bond formed between G216O and L86N1, L86H9. (**C**) the hydrogen bond formed between S214O and L86N4, L86H13.

We further analyzed three hydrogen bonds between thrombin and L86 present in X-ray structure, as shown in Fig. 4A. The time evolutions of the hydrogen bond lengths between thrombin and L86 during MD simulation in both force fields are shown in Fig. 5. After a long time MD simulation, we unexpectedly observed that two important hydrogen bonds between L86 and GLY216 residue were broken after approximately 190 ns using AMBER12SB with occupancies of 34% and 34%, respectively. Our simulation time used in the present study was sufficient enough to observe this important phenomenon. However, these two same hydrogen bonds were well preserved under PPC, with occupancies of 95% and 93%, respectively, and although these hydrogen bonds were broken after a short period of time (from 200 to 230 ns), they quickly reformed and then remained stable in the following time. The hydrogen bond between SER214 and L86 was well preserved in the simulations, with average bond lengths similar to the experimentally measured value, regardless of the force field employed. However, the occupancy of the hydrogen bond under PPC (99%) was higher than that under the AMBER force field (94%), suggesting these hydrogen bonds were more stable or energetically stronger.

**Figure 6:**
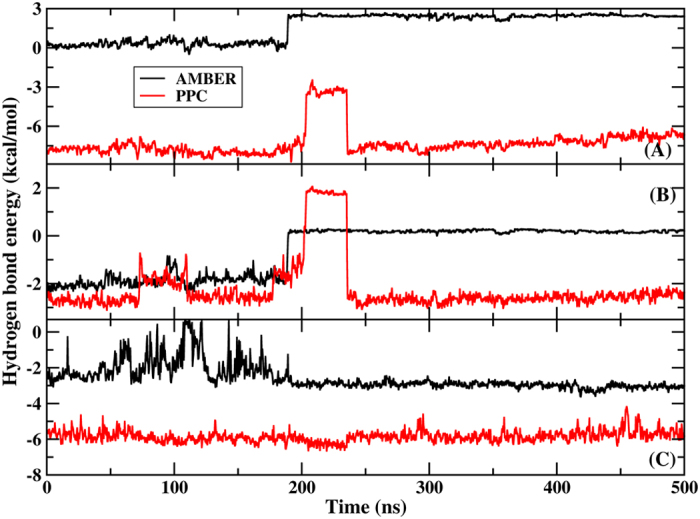
Evolution of the hydrogen bond energy between thrombin and L86 under AMBER (black) and PPC (red). (**A**) The energy of the hydrogen bond formed between L86O1 and G216N, G216H. (**B**) The energy of the hydrogen bond formed between G216O and L86N1, L86H9. (**C**) The energy of the hydrogen bond formed between S214O and L86N4, L86H13.

To further compare these differences in detail, the final structures produced by the MD simulation are also shown in Fig. 4B,C for AMBER and PPC, respectively. Only one hydrogen bond was preserved between thrombin and its ligand under the AMBER force filed (Fig. 4B), whereas three hydrogen bonds were stable using PPC (Fig. 4C). Furthermore, the protein secondary structure, including the GLY216 residue, underwent incorrect deformation from a coil to a β-sheet, and the ligand structure also exhibited a large deviation from the native structure under the AMBER force field. However, this phenomenon was not observed under PPC. We calculated the three hydrogen bonds energies during MD simulation, and as shown in Fig. 6, these values were different. Under the AMBER force field, two hydrogen bonds were broken during MD simulation, and thus, they had positive energies. In contrast, under PPC, the two hydrogen bond energies were approximately −7.27 and −2.33 kcal/mol (Fig. 6A,B), respectively. The energy of the hydrogen bond between L86 and SER214 was 3.23 kcal/mol stronger in PPC simulation (average hydrogen bond was −4.50 kcal/mol) than in the AMBER simulation (average hydrogen bond was −1.55 kcal/mol). Because of the lack of electronic polarization, the calculated electrostatic energies of hydrogen bonds were relatively weak and even positive using the AMBER force field. Thus, the results of this study highlight the importance of the electrostatic interactions in stabilizing the hydrogen bonds between proteins and ligands.

The MM/PBSA method was used to calculate the binding free energies of the complex, and the calculated binding free energies from 200 snapshots extracted from MD trajectories were averaged. In this calculation, the binding free energy is split into five components: electrostatic, van der Waals (vdW), polar solvation energy, non-polar solvation energy and entropy loss. The decomposed energies, determined as the total binding free energy from the MM/PBSA calculation and the evaluation the standard deviations of the calculation are listed in Table 1. Under PPC, the computed electrostatic interaction energy (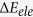) was lower than that obtained using the AMBER force field. The mean electrostatic contribution to the free energy was −27.76 kcal/mol in PPC, which was 12.75 kcal/mol stronger than that under the AMBER force field (−15.01 kcal/mol). L86 forms three stable hydrogen bonds with thrombin; thus, the electrostatic interaction was strong in the PPC MD simulation. However, two of these three hydrogen bonds were broken in the AMBER simulation, reflecting the weak interaction energy. The vdW interaction energies contributed −53.59 and −52.42 kcal/mol to the binding affinity using AMBER and PPC, respectively; these energies represented the primary driving force for L86 binding to thrombin in both force fields. Although L86 generated strong electrostatic and vdW interactions with thrombin, these favorable interactions were partially canceled by unfavorable polar solvation energies (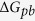) of 47.00 and 47.90 kcal/mol for AMBER and PPC, respectively. The non-polar solvation term contributed −5.19 and −4.95 kcal/mol, which were slightly favorable values, and the entropy loss reduced the binding strengths by 23.10 and 24.19 kcal/mol for AMBER and PPC, respectively.

**Figure 7:**
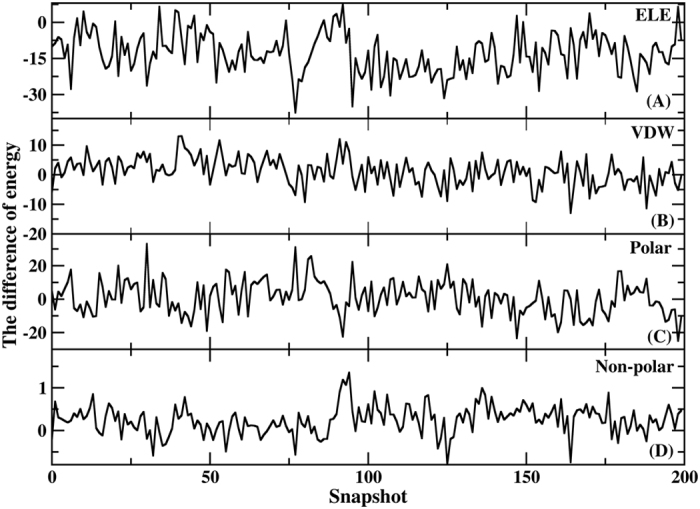
The difference in the binding free energy components from 200 snapshots between PPC and AMBER (**A**) Electrostatic energy term, (**B**) vdW term, (**C**) polar solvation energy term, and (**D**) non-polar solvation energy term.

Moreover, the total binding free energy calculated using the AMBER force field was −3.70 kca/mol, which is substantially different from the experimentally observed value. The binding free energy predicted using PPC was −13.04 kcal/mol. In view of the theoretical errors in calculated binding free energy, the theoretical result is consistent with the experimental result of Nantermet *et al*. (−13.70 kcal/mol)^80^. The differences in the binding free energy components of PPC and AMBER determined from 200 snapshots are shown in Fig. 7. Only Fig. 7A exhibited a difference, reflecting the electrostatic contribution, which fluctuated from −37.58 to 7.66 kcal/mol. The differences in the other terms were generally small, even negligible. Comparing the results from the AMBER force field with those from the PPC calculation revealed that the difference in the final binding free energy primarily reflected differences in the calculated electrostatic contributions. Thus, the inclusion of electronic polarization is essential for studying the interaction mechanisms of protein-ligand binding. The missing polarization effect in the AMBER force field underestimates the importance of the electrostatic contribution to the binding free energy.

Force field involving the polarization effect has substantially improved the performance of MD simulation, resulting in more reasonable protein-ligand structural ensembles. The results of MM/PBSA calculations based on the trajectory of the PPC simulation and using the PPC charge to evaluate the electrostatic energy were more consistent with the experimental data than those of the AMBER force field. Thus, we performed another MM/PBSA calculation in which the complex structure was extracted from the PPC simulation but using standard AMBER charge to evaluate the binding affinity. A shown in Table 1, the electrostatic contribution was lightly stronger than that from the AMBER trajectory, and the vdW energy and non-polar solvation free energy were independent of the charge model. This gave a total binding free energy of −3.66 kcal/mol, which was inconsistent with the experimental data. The mixed charge calculation indicated that unpolarized AMBER charge did not capture the binding free energy, despite its accurate structural ensemble. In the other hand, we used the AMBER simulation trajectory with the PPC charge to calculate the binding affinity. As shown in Table 1, the binding free energy of was −0.40 kcal/mol. Although the electrostatic energy was accurately captured using PPC, the complex structural ensemble generated from the AMBER simulation was unreliable, reflecting the lack of polarization. We also computed the binding free energies of the X-ray structure using AMBER and PPC. PPC generated a value of −14.04 kcal/mol, consistent with the experiment value, whereas AMBER resulted in a value of is −2.20 kcal/mol. The result obtained with PPC was also consistent that from the PPC trajectory, suggesting that the protein structure did not change much during simulation. Similarly, the electrostatic contribution dominated differences in the binding free energy of the X-ray structure between AMBER and PPC, as shown in Table 1, and the PPC computation generated much lower values than those obtained from AMBER. The non-polarizable AMBER force field did not adequately describe the binding affinity of the thrombin-L86 complex. Thus, including electronic polarization is essential in MD simulation and binding free energy calculation.

**Figure 8:**
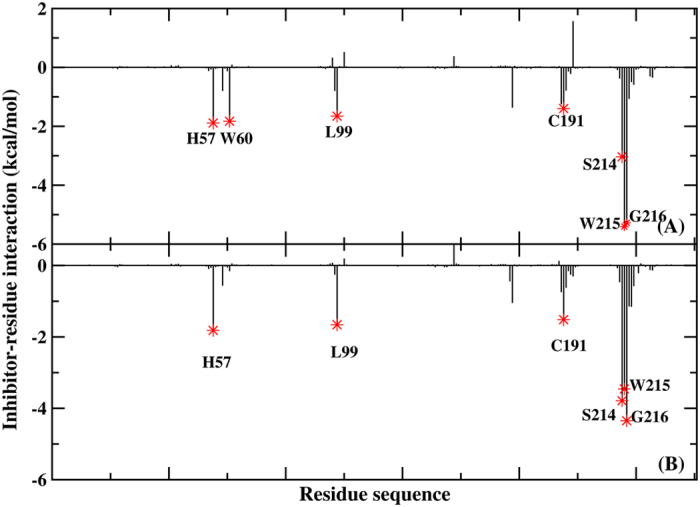
Ligand L86-residue interaction spectrum, (**A**) X-ray structure, and (**B**) averaged structures over the PPC MD based on collected snapshots.

**Figure 9:**
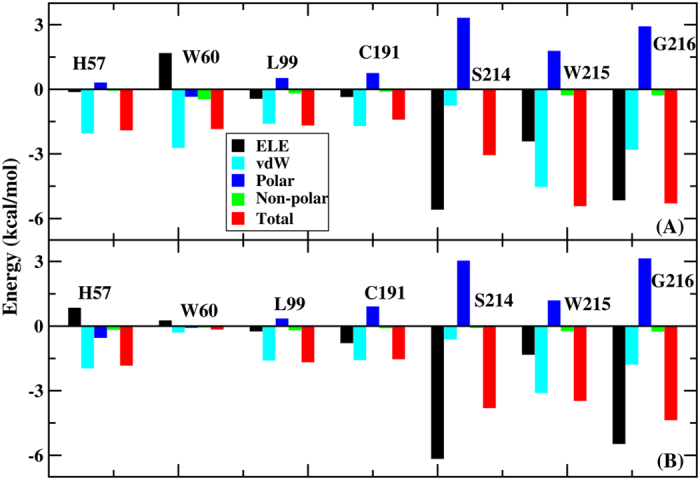
Decomposition of the binding free energy on a per-residue basis into contributions from electrostatic interactions, vdW energy, polar solvation energy, and non-polar solvation energy for residues with the binding energies exceeding 1.4 kcal/mol. (**A**) X-ray structure and (**B**) averaged structure for the PPC MD based on collected snapshots.

Because of the failure to predict the binding free energy in the AMBER force field, the contribution of each residue to the binding was calculated for X-ray and simulated structure using PPC to further investigate the dynamics and fluctuation of the thrombin-L86 complex. Figure 8 compares L86-residue interaction spectrum of the X-ray structure and the average of the 200 snapshots extracted from the PPC simulation. Clearly, the two interacting spectra were quite similar with a correlation coefficient of 0.93. Although a slight difference was evident between the two spectra, the simulation result was reasonable because the MD can relax the X-ray structure to obtain a stable conformation. As shown in Fig. 8, the dominant binding attractions were determined by seven residues—HIE57, TRP60, LEU99, CYS191, SER214, TRP215 and GLY216—which contributed more than 1.4 kcal/mol in the X-ray structure. In the simulated structure, the primary bonds involved HIE57, LEU99, CYS191, SER214, TRP215, and GLY216. Decomposing the binding energy into different energy terms on a pre-residue basis, the electrostatic energy, vdW, polar and non-polar solvation energy terms of the above seven residues were determined from the X-ray and simulated structures, as shown in Fig. 9, respectively. The results showed that the vdW and non-polar solvation energies were favorable for binding. No obvious differences between the two spectra of the above residues were observed, except for TRP60. The vdW interaction was the primary driving force for the HIE57, LEU99, CYS191 and TRP215 residues. L86 formed not only C-H···π interactions with the imidazole of HIE57 and the alkyl groups of LEU99 and CYS191 but also N-H···π stacking interactions with the peptide bond between TRP215 and GLY216. Thus, a strong hydrophobic interaction existed between L86 and these residues. Additionally, SER214 and GLY216 formed hydrogen bonds with L86, producing a strong electrostatic interaction. For TRP60, the binding energy was apparently stronger in the X-ray structure than in the simulated structure. This increased energy was primarily attributable to the vdW interaction energy, which was 2.43 kcal/mol higher in the X-ray structure than in the simulated structure. The indole of TRP60 forms a C-H···π interaction with the alkyl of L86 in the X-ray, but this residue was positioned far from L86 in the simulated structure.

## Conclusions

Because thrombin is important in biology and medicine design, this enzyme has become the focus of one of the most extensive studies of all proteases. In this study, large-scale MD simulations in explicit water were carried out for 500 ns to characterize thrombin and the ligand binding affinity under nonpolarizable AMBER and polarizable PPC force fields. PPC is derived from the quantum mechanical calculation for a protein in solution using the molecular fractionation with conjugate caps (MFCC) approach combined with the continuum dielectric model for the solvent in a self-consistent treatment. Because PPC explicitly includes the electronic polarization effect of a protein at a given structure, this strategy provides more accurate electrostatic interactions than typical force fields. And the time scale of simulation is sufficient to reliably quantify sample the conformational space. The results of this study showed the following features:

1. Under nonpolarizable AMBER force field, the numbers and energies of hydrogen bonds in both intra-thrombin and thrombin-ligand were smaller and weaker than those generated through the polarizable PPC force filed.
2. Two important hydrogen bonds between thrombin and its ligand were broken under AMBER MD simulation after 190 ns. In contract, two same hydrogen bonds were well preserved under PPC simulation of 500 ns.
3. The thrombin-ligand structure was more dynamically stable under PPC force field than AMBER force filed.
4. The calculated binding free energy was consistent with the experimentally measured value under PPC force field. Because of the broken hydrogen bonds, the standard AMBER force field underestimated the importance of electrostatic contributions to thrombin-ligand binding, therefore, did not adequately capture the binding affinity.
5. Furthermore, directly comparing the PPC simulation result with experimental data revealed that L86 was well accommodated in the binding pocket and showed strong electrostatic and vdW interactions with thrombin.

The results of this study demonstrated that the polarization effect is essential in MD simulation and plays a critical role in protein-ligand binding.

### Computational Method

The initial structure of thrombin and L86 was downloaded from Protein Date Bank (entry: 1NM6). The MD simulations were performed using the AMBER12SB and PPC for 500 ns. When preforming the PPC simulation, other than the atomic charges, the force field parameters were the same as those in the AMBER12SB simulation. All hydrogen atoms were automatically added using the Leap module in the AMBER package. The protein-L86 complex was socked in a periodic box of TIP3P water^83^, and the minimal distance between the protein and the boundary of the box was set to 12 Å. Four chloride ions were added to neutralize the system. Then, the system was relaxed using a standard produce: First, only the solvent molecules were free to move, while the protein and ligand atoms were constrained by an external force. Second, the entire system was optimized until convergence was reached. Subsequently, the system was gradually heated to 300 K over 300 ps and further relaxed for 500 ns using the NPT ensemble without any restraints on solute atoms. The integral time step was set to 2 fs. The temperature was regulated with Langevin dynamics heat coupling scheme^84^, and the collision frequency was set to 1.0 ps^−1^. The SHAKE algorithm^85^ was employed to restrain all covalent bonds involving hydrogen atoms. Long range electrostatic interactions were calculated using the particle mesh Ewald (PME) method^86^, and the vdW interactions were truncated at 12Å. Last, 200 snapshots were extracted along the MD trajectory after equilibrium to calculate the binding free energy using the MM/PBSA program in AMBER12, and normal mode analysis was used to calculate the entropy change. Because the entropic calculation for a large system is extremely time consuming, 20 snapshots were used to estimate the contribution of the entropy to decrease the computational time.

A detailed description of the derivation of PPC had been previously published^39^. Thus, only a brief review of the method is given here. In the PPC approach, the entire protein was decomposed into amino acid-based fragments through molecular fragmentation using a conjugated caps scheme^87^ to achieve the electronic structure of the protein through fully quantum mechanical calculations. Subsequently, using a restrained electrostatic potential (RESP) procedure, the calculated electron density was used to fit the atomic charges. The Poisson-Boltzmann (PB) equation for protein in solvent was used to solve the self-consistent reaction field equation using the DelPhi program^88^ to generate discrete surface charges at the protein-solvent interface. In solving the PB equation, the probe radius, which was used to calculate the solvent accessible surface, was 1.4 Å, and the grid density used was 4.0 grids/Å. The dielectric constant was set to 1.0 for the protein environment and 80.0 for the solvent. The surface charges, which mimicked the solvation effect, and the charges from other protein fragments were considered as background charges in the next cycle of the quantum mechanical calculation for protein fragments. These steps were reiterated until convergence was reached; typically, convergence was realized after 4–6 iterations. All quantum mechanical calculations were performed at the B3LYP/6-31G^*^ level.

The binding free energy of the protein-ligand was evaluated using MM/PBSA, a post-processing method, in a relatively computationally efficient manner. In the calculation, the binding free energy (ΔG) is represented as:


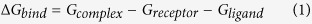


where 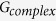,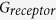, and 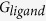
represent the energies of the complex (thrombin/L86), thrombin and L86, respectively. The free energy of each of these parameters is calculated as the sum of the following four terms:


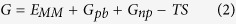



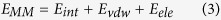



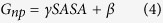


where
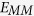
is the molecular mechanics free energy in the gas phase, including the internal energy of the molecule, and the electrostatic and vdW interactions (Equation (3)). Because the binding energy was calculated from a single trajectory, the internal energy difference is equal to zero. 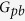
and 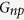 represent the polar and non-polar solvation free energy terms. 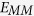 is calculated using the sander module, and 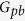 is calculated by solving the PB equation using the PBSA module of the AMBER suite. The interior and exterior dielectric constants were set to 1 and 80, respectively. The non-polar free energy (Equation (4)) is calculated by the empirical solvent-accessible surface area (SASA) formula using the MSMS program^89^, and the surface tension γ and offset β are empirical constants of 0.00542 kcal/(mol·Å^2^) and 0.92 kcal/mol, respectively. TS is a term involving the entropy effect, approximated from normal mode calculation using the NMODE module^90^ in AMBER.

## Additional Information

**How to cite this article**: Duan, L. L. *et al*. Large-scale molecular dynamics simulation: Effect of polarization on thrombin-ligand binding energy. *Sci. Rep*. **6**, 31488; doi: 10.1038/srep31488 (2016).

## Figures and Tables

**Table 1:** The decomposition of the binding free energy (kcal/mol).

Charge	Trajectory	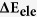	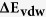	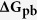	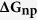	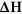	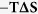	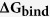	Exp*
AMBER	AMBER	−15.01 ± 5.9	−53.59 ± 5.5	47.00 ± 7.2	−5.19 ± 0.2	−26.80 ± 7.9	23.10 ± 4.4	−3.70	−13.70
PPC	PPC	−27.76 ± 5.8	−52.42 ± 4.4	47.90 ± 7.2	−4.95 ± 0.3	−37.23 ± 7.4	24.19 ± 5.8	−13.04	
AMBER	PPC	−18.75 ± 4.4	−52.42 ± 4.4	46.85 ± 6.2	−4.95 ± 0.3	−29.26 ± 6.4	25.60 ± 5.0	−3.66	
PPC	AMBER	−15.53 ± 9.0	−53.59 ± 5.5	52.15 ± 8.4	−5.19 ± 0.2	−22.15 ± 10	21.75 ± 4.9	−0.40	
AMBER	X-ray	−22.18	−62.18	65.00	−5.26	−24.62	−22.42	−2.20	
PPC	X-ray	−31.81	−62.18	59.95	−5.26	−39.30	−25.26	−14.04	

All values are shown in kcal/mol; Errors labeled by the signs ± represent the standard deviations. *The experimental binding free energy is calculated using K_i_, according to Nantermet *et al*.^80^.
